# Discussion on Dr. Wallace’s Paper*Taken entire from the Dallas News’ report, in its issue of April 25th, as we have not been able to get a copy of the Association stenographer’s report in time for this edition.—Ed.

**Published:** 1895-05

**Authors:** 


DISCUSSION ON DR. WALLACE'S PAPER.*

There was a ripple of excitement throughout the hall during Dr. Wallace's closing remarks. As soon as he had finished, there was a loud burst of applause, and Dr. S. H. Stout, of Dallas, sprang to his feet. Dr. Stout made a long and vigorous argument, which was frequently interrupted by applause. He said that he could not agree with Dr. Wallace in regard to the latter's views in comparing the Chinese and the American people. There is a reason why among the Chinese there is not so much insanity as among us, but it is not the same reason as Dr. Wallace explained. With the civilization of Europe and America, and again when Columbus discovered this country, a new spirit of adventure was awakened. It developed a wide-awake people, who were disposed to make money, by legitimate trade if possible, and if not, to make it by piracy and robbery. To-day the same state of affairs almost ruled. In the inordinate desire of greed for gain we overlooked the rules of hygiene, and often overburdened our minds with hopes of financial gain which did not pan out. The adventurers mentioned had a tendency to override the spirit of Christianity. I don't think that Christianity itself is anywhere at fault, but the progress of the times has been the cause of departing, in a great measure, from the spirit of Christianity. While the Chinese are believers in Confucius' religion, and we are believers in the Biblical religion, we can not attribute insanity any more to our belief than to theirs. I think that in ninety-nine cases out of one hundred insanity is due to a too intense occupation of the mind. The Chinese do not aspire to civilization, while we have no end to our aspirations in this cause. I believe that normal psychology should be introduced into the medical schools of the world, and that the medical fraternity should be taught its doctrines, as much as they are taught any others.

Dr. O. L. Williams, the next speaker, said he agreed with Dr. Wallace, except that he believed Dr. Wallace rather overstrained the religious condition with the pathological condition. Dr. Williams said he believed Dr. Clopton, in his paper on physical culture, had struck the key note of the preventive of insanity. He admitted that there was an increase in the number of insane patients, and said that the remedy was to begin at the cradle.

---

*Taken entire from the Dallas News' report, in its issue of April 25th, as we have not been able to get a copy of the Association stenographer's report in time for this edition.—ED.

The wave of intemperance in liquors, in cocaine, in morphine, and all that class of stimulants, is the result of unsystematical development of the human organs. The remedy lay in physical culture early in youth.

Dr. G. C. Head spoke next. He said he was loath to attack the paper, or to make any violent criticisms of it, on account not only of the age of the author, but also on account of its great literary skill, which he considered wonderful. At the same time he was not prepared to stand by and indorse the sentiments, or to have them disseminated among the medical profession at large as receiving an indorsement from this Association. If the paper is to be construed as a direct or indirect attack upon the great moral and Christian institutions of our nation, the Texas State Medical Association can not afford to indorse it. The Texas State Medical Association can not afford to let the paper go out among the young people of the State.

Dr. A. G. Clopton next arose. He expressed his entire disapproval as to the gist of the paper. The paper itself seemed to give to the Christian religion the cause of insanity among our people. I deny that. (Applause.) When the paper was being read, my mind was carried back to a number of cases within my knowledge that had been carried to an asylum and to private institutions, and among them all I could not point to a single minister of the gospel. (Applause.) Nor do I remember the instance of one ever going insane. (Renewed applause.) I myself am a church member (applause), and in looking over the number of church members with whom I have been associated, I do not recall one from the living church of God who has gone to an insane asylum. (Applause.) I admit that I have known persons who have gone insane through religious fanaticism, but Dr. Wallace's paper can not be applied to Christianity at large on that account. I wonder how Newton ever kept out of an asylum, for he was certainly insane, so we have just been told. Yes, and I wonder why the venerable Gladstone is not in a madhouse, according to Dr. Wallace's theory, for his is the living embodiment of the Christian faith.

Loud applause greeted the conclusion of Dr. Clopton's remarks.

Dr. A. H. Schenk spoke next. He said: "It has come to my mind that the causes of degeneration so noticeable to-day are the effect of the literature of the times on the minds that are now developing. I have recently seen the statement that it would not be long until a woman who read books like 'Trilby' and 'The Heavenly Twins,' would give birth to devilish triplets. (Applause.) A line of writers has recently developed who take up pathological and psychological subjects and treat with them. Their writings show they are not prepared to do justice to the subject. In 'Trilby,' for instance, and other books on the subject of hypnotism, there is a great misconception shown on the part of the writers on one subject over another. The writer of 'Trilby' and the writers of other novels of a like character make things appear natural that are not true. I think the literature of the times has a tendency to produce a very evil effect on the mental and physical growth of young persons."

Dr. J. W. Carhart, who waxed warm in his argument, spoke next. He said: "I do not wish Dr. Wallace's paper to pass without putting in my protest. The paper shows a decided disadvantage to the religion of Jesus Christ as compared with that of Confucius. I admit that there is less insanity in China than in America, but at the same time I insist that the Indians, the aboriginal inhabitants of this country, were not insane. We ought to be cautious how the sentiments of this paper are indorsed by the Texas State Medical Association. We do indorse the religion of Jesus Christ and not of Confucius. (Applause.) The venerable Dr. Stout has given us the key to the increase of insanity in this country—the rush for wealth—and he has also given us the origin of this. This continues in spite of the elevating, the refining, the ennobling, the dignifying influence of the Christian religion, but the time is coming when the religion of Jesus Christ will prevail over that of Confucius, and the cross will surmount the crescent or any other emblem of paganism or outside creeds. (Applause.) This is no time and no place for the Texas Medical Association to pronounce the religion of Jesus Christ a failure (loud applause) and to work for a new religion. We, as members of the Texas Medical Association, are not here to do that, and I simply want to enter my protest against our doing that. The literature, the frivolities, the vices and intemperance in various forms in this country are responsible, in my mind, to a great extent for insanity and the many nervous affections which we, as a people, are suffering from to-day."

As the speaker sat down, Dr. Wallace arose and walked to a place beneath the President's desk. He faced the audience. He was visibly affected by the remarks made. He said: "I had no idea I had said one word that could, by any possible construction, be interpreted as arraigning Christianity. I have as great a veneration for the name of Jesus Christ as has Dr. Clopton or the presiding officer. (Applause.) I have as high a conception for the morals taught by the Savior as these gentlemen. I arraigned theology and said not a word about Christianity, and defy you to show me one word in the paper that arraigns Christianity. I do not care whether the paper is published or not; it would not derogate anything from me nor add anything to me, but I wish to say to the young men of the Association that things have come to a fine pass when a man who has been for twenty-seven years a member of this body, who had more to do with its organization than any other man, who had tried to live a blameless life, should be arraigned before this Association as the author of a paper against the Christian religion. I am against theology. I believe it is the curse of the country, but at the same time I have given forth no expressions against the meek and lowly Jesus or his teachings. On the contrary, I especially tried to make myself distinctly understood as indorsing him. I said not one word against the Savior, nor do I feel it in my heart; but, gentlemen, is the Christianity of these gentlemen (here the doctor waved his hand in the direction of Drs. Clopton, Head and others) so ready to topple over that it won't bear the least insinuation or discussion? (Applause.) If it is, it is in a bad way, surely. As for Dr. Clopton, or any one else indorsing me, I don't want their indorsement. I like to meet with the approbation of my fellow-men, but if I must do it at the expense of stultifying myself, I can not do it. (Applause.) What I have said, I am willing to face at the judgment seat. (Applause. I take it as exceedingly unkind that any of these gentlemen should have seen fit to arraign me here on this occasion. They have arraigned a man, to say the least, who means as well as they do." (Applause.)

As soon as Dr. Wallace had finished, a motion was made that his paper be printed in the proceedings. There were a dozen seconders. The Secretary explained that the usual mode of procedure was to refer papers to the Publishing Committee. The first motion was so amended. The chair put the motion, and yeas and nays seemed evenly divided. There were loud calls for a division. The chair requested those in favor to rise, while the Secretary counted them. Only thirty-two stood up. There were fully over one hundred members present. When those against the motion rose, the Secretary could only count ten. Dr. Clopton, and the majority present, did not vote. The paper was referred to the Publishing Committee by a majority of twenty-two. The session closed shortly afterward, at 1 p. m., adjourned until 2:30 p. m.

The JOURNAL is surprised and regrets to learn, as we do, by a letter from Secretary West, that the Publishing Committee decline to incorporate the paper in the annual volume of Transactions.

